# Tools and methods for cataract recognition in low-resource settings: A narrative review

**DOI:** 10.1371/journal.pgph.0004619

**Published:** 2025-05-16

**Authors:** Paulina N. Truong, Elizabeth A. Merlinsky, Nicholas C. Maamari, Christina Y. Weng, Andrew G. Lee

**Affiliations:** 1 School of Medicine, Baylor College of Medicine, Houston, Texas, United States of America; 2 Department of Ophthalmology, Baylor College of Medicine, Houston, Texas, United States of America; 3 Department of Ophthalmology, Blanton Eye Institute, Houston Methodist Hospital, Houston, Texas, United States of America; 4 Department of Ophthalmology, University of Texas MD Anderson Cancer Center, Houston, Texas, United States of America; 5 Departments of Ophthalmology, Neurology, and Neurosurgery, Weill Cornell Medicine, New York, New York, United States of America; 6 Texas A&M College of Medicine, Bryan, Texas, United States of America; 7 Department of Ophthalmology, The University of Iowa Hospitals and Clinics, Iowa City, Iowa, United States of America; 8 Department of Ophthalmology, University of Texas Medical Branch, Galveston, Texas, United States of America; NSCB Government Medical College Department of Surgery: Netaji Subhash Chandra Bose Medical College and Hospital Department of Surgery, INDIA

## Abstract

Cataracts are a leading cause of global blindness, with many low-resource populations having poor access to eye care. While much is known regarding surgical access and outcomes, less is understood about the initial detection of operable cataracts in low-resource settings. We performed a comprehensive literature review on existing and emerging cataract screening methods in low- and middle-income countries (LMICs). The search keywords were “cataract detection, diagnosis, assessment, and evaluation in low- and middle- income countries”. 1,825 articles were identified and 62 were selected for final inclusion comprising reviews, meta-analyses, and original research studies. Only 12 studies proposed new cataract screening methods for low-income settings. We extracted data on the efficacy and cost-efficiency of these novel methods and compared them to existing conventional cataract diagnosis methods. 8 out of 12 original research works developed novel devices or imaging modalities such as low-cost ophthalmoscopes and retinal cameras for cataract detection. 7 studies leveraged non-ophthalmologist staff to test their methods, suggesting a potential benefit of employing additional personnel for cataract screening. 8 studies utilized tele-ophthalmology and artificial intelligence (AI) platforms with high cataract detection accuracy. Overall, rates of cataract diagnosis in LMICs can be increased through a multifaceted strategy involving low-cost, portable devices operated by local healthcare workers and augmented by telehealth or AI approaches. Such efforts can aid in addressing the disparity between cataract diagnosis and surgical intervention in low-resource environments. However, improvements in research infrastructure are needed to support such efforts and the ability to thoroughly evaluate new innovations.

## Introduction

Cataract has been identified as the leading cause of blindness worldwide in adults aged 50 years and older [1]. The aging global population has led to increased rates of age-related cataract [2–5]. Cataracts are responsible for over 50% of all blindness in most developing countries [4] and have been deemed the second most unevenly distributed cause of blindness worldwide, with higher disability-adjusted life years (DALY) in Oceania, South and Southeast Asia, the Eastern Mediterranean, and Africa [6–8]. Many of these areas are home to populations that are at a particularly high risk of developing cataracts earlier in life due to higher levels of ultraviolet exposure, pollution, and differences in dietary habits [9–11]. While major progress has been made to address certain challenges in global cataracts, such as expansion of intraocular lens surgery as a whole [12], identifying and implementing strategies to improve the quality, productivity, equity, and access of cataract services remains the second out of sixteen Grand Challenges in global eye health [13].

The disparity in cataract treatment in low- and middle-income countries (LMIC) may be explained by multiple factors. Low-income and low-resource or rural settings have been shown to have lower densities of ophthalmologists, with ratios around 1 ophthalmologist per 1 million people in LMICs, compared to 200 ophthalmologists per million people in the US or Western Europe [3–5,14–18], and primary care workers are poorly equipped to evaluate vision without the proper tools and knowledge regarding ophthalmic disease. One 2020 study in Nepal demonstrated that only 14% of primary health care workers were trained to measure visual acuity, only 5.7% had a distance vision chart, and even fewer had ophthalmic equipment such as a torch [19]. Other barriers to eye care in low resource settings may include cultural perceptions of eye disease, lack of transportation to clinics that provide eye care, and poor surgical outcomes [5,15,16,20,21].

Despite the global inequity related to cataracts and their treatment, cataract surgery in LMICs has been shown to be highly cost-effective, typically costing less than $150 but as low as $9 per DALY [22–25]. Approaches to further increase the cost-effectiveness of cataract management without jeopardizing patient safety, include omitting visits on post-op day 1 in case of uneventful surgeries [26]. There exists a growing base of research surrounding surgical barriers, access, techniques, and outcomes in LMICs [27,28]. However, relatively little is known about diagnostic methods in these settings, and population-based surveillance of cataract surgical needs has been identified as an unmet need [29]. Overall, the paucity of research in this area illuminates a need for an improved referral-to-surgery pathway. We review the literature on novel tools and methods for cataract recognition in low resource settings and identify gaps in the evidence base for future research. We define novel methods as non-standard tools (i.e., other than the slit lamp biomicroscope, direct, or indirect ophthalmoscope) or systems for eye care that are not yet reported in the literature for cataract screening.

## Methods

A literature search from 1965 to 2025 was performed in PubMed, Cochrane, and Embase using the following search terms: (cataract) AND ((detection) OR (diagnosis) OR (evaluation) OR (assessment)) AND ((global) OR (low resource) OR (low middle income) OR (third world) OR (developing country)). Our search returned a total of 1,825 results. Articles were initially screened by title and abstract and were included if they described disparities, availability, or costs of eye care and/or cataracts in low-resource settings (i.e. LMICs or rural areas in high-income countries) or proposed a novel diagnostic modality or model of eye care delivery for cataract diagnosis in low-resource settings. Articles were excluded if they did not meet the above criteria, described a disease other than cataract, did not propose a new intervention to diagnose cataract, described an intervention that was not novel, proposed an intervention that was not developed, tested, or intended for applications in low-resource settings, only described surgical methods, access, or outcomes, or were non-human studies. This initial screening resulted in 269 titles for a second round of full-text analysis, with a final selection of 62 articles. Out of these, 27 were reviews and meta-analyses, and the remaining 35 were original research. Papers related to the costs or disease burden of cataracts were excluded from our official analysis that was restricted to methodological papers describing the application of new technologies for cataract screening. Based on the results of our search query, we identified 12 original papers that developed and tested novel methods to diagnose cataracts in LMICs; these papers were analyzed with respect to their context, efficacy, and applications (**Table 1**).

**Table 1:** Original interventions to increase accessibility & decrease costs of cataract screening in LMICs.

Paper	Country	Cohort Size	Proposed Intervention	Personnel Tested	Efficacy/Key Findings	Cost	Applications
Devaraj 2024	India	13,633 smartphone images for testing, 604 images for testing	Artificial intelligence web/mobile app for automatic cataract detection using smartphone camera	Accredited Social Health Activists (frontline community health workers)	Overall 86.6% SEN, 93.3% SPE, 58.4% PPV, 98.5% NPV, 89.9% ACC	N/A	Population-level screening tool adaptable to variable image types/quality and accessible to frontline healthcare workers to reduce cataract backlog
Gan 2023	China	647 slit-lamp anterior segment images	Artificial intelligence platform for diagnosis & referral of cataracts using mobile camera images	N/A	Automatic segmentation takes 2m43s, with training and test set ACC of 95% and 85% respectively for classifying incipient, intumescent, mature, and hypermature cataracts. PA, IoU, Dice coefficient improved by 8.4, 14.9, and 9.5% compared to previous methods. 96% AUC compared to 97% AUC for manual segmentation method	N/A	Cost reduction & medical settings with insufficient ophthalmic resources
Pathak 2022	India	>200 training digital color images, > 300 test patients	Portable image capture device + cloud-integrated cataract detection/grading algorithm	Unknown image capturer (“doctor”/ “eye expert”)	98% ACC	N/A	Low cost, simple device; simpler algorithm compared to deep learning models; mountable, non-invasive, non-mydriatic nature of device does not require in-person screening
Wu 2022	China	14,600 cataract & non-cataract fundus images for training, 1800 for testing	Artificial intelligence platform for cataract diagnosis using fundus images	N/A	>91% AUCs, > 84% ACCs, > 71% SENs, and >89% SPEs for categorizing cataract, non-cataract normal quality images, and non-cataract poor quality images. Anti-interference feature improves performance by 10%	N/A	Accurate cataract screening even with the interference of poor-quality images
Katibeh 2020	Iran	2,520 participants	Smartphone camera with Peek Retina fundoscopy software/attachment and mobile health application	Primary health care workers	18.0% of patients were identified with cataract; eye care utilization after mobile health screening was 2.9% higher than conventional screening	N/A	Accessible device for non-ophthalmologists to improve eye care utilization at the population level
Wu 2019	China	37,638 slit lamp training images	Artificial intelligence platform for diagnosis & referral of cataracts using mobile camera images	N/A	>99% AUC for detection of normal lens, cataract, & postoperative eye	N/A	Allows for management & immediate referral to secondary/tertiary levels of care in areas with no primary eye care services
John 2015	India	19,634 participants	Real-time fundus image sharing between remote villages & ophthalmologists in city hospital	Unknown image capturer	15.3% of patients were identified with cataract	N/A	Live access to ophthalmology care in rural areas with limited connectivity
Rahman 2013	United Kingdom	73 test patients (122 eyes)	Handheld LED-powered device for assessment of red reflex	2 nurses examined all patients independently	Advanced cataract: 86–93% SEN, 95% SPE, 0.81-0.88 κ for agreement with slit lamp, 0.74 inter-observer agreement. Mild cataract: 71–85% SEN, 95% SPE, 0.61-0.80 κ for agreement with slit lamp, 0.61 inter-observer agreement.	70% cost reduction compared to USA direct ophthalmoscope, > 97% reduction compared to slit lamp	Low cost materials, easy to use device for non-ophthalmologists
Shahid 2012	USA	341 participants	Retinal camera with high-speed Internet for off-site image evaluation	Ocular imaging specialist (capture images), optometrist (analyze images, refer to specialist)	21% of patients were identified with cataract	N/A	Sensitive detection of eye conditions in high-risk groups with low access to eye care
Harle 2007	United Kingdom	145 participants	Optyse direct ophthalmoscope without a lens focus system	6 optometrists	14% of patients identified with cataract. Reduced clarity of view in evaluating cataracts compared to ophthalmoscope. Low inter-observer variability & variability with conventional ophthalmoscope	37-94% cost reduction compared to UK direct ophthalmoscopes	Low-cost alternative to direct ophthalmoscope
Pon 2005	New Zealand	18 providers, 68 patients	Handheld pen torch ophthalmoscope	5 pediatricians/ pediatric registrars, 4 midwives, 3 ophthalmologists, 3 NICU nurses, 2 GP, 1 optometrist	68% SEN, 72% SPE, 71% PPV, 70% NPV for assessment of retinal red reflex. These values are -7%, + 9%, + 5%, and -3% compared to direct ophthalmoscope	94% cost reduction compared to NZ direct ophthalmoscope	Evaluation of adult and pediatric/congenital cataracts in low-income settings
van Dijk 1999	Malawi	163 participants	Visual function questionnaire surrounding activities of daily living	1 unknown surveyor	Visual function scores correlate with visual acuity, contrast sensitivity, near vision, self-reported visual problems. Mean visual function score of 35.1/100	N/A	Simple tool usable by community workers to increase awareness of visual problems & encourage surgical uptake

ACC = accuracy, PA = pixel accuracy, IoU = intersection over union, AUC = area under ROC (receiver operating curve) curve, SPE = specificity, SEN = sensitivity, PPV = positive predictive value, NPV = negative predictive value.

## Results

In ten out of twelve original studies, the authors developed low-cost, portable devices that were either newly developed specifically for cataract diagnosis or novel applications of existing devices in low-resource settings. These include an LED-powered ophthalmoscope, pen torch ophthalmoscope, lens-free ophthalmoscope, smartphone cameras, and retinal cameras [15,30–37]. Specifically, four papers suggested the usage of mobile cameras to capture ocular images which are then analyzed automatically [32,33,35,37]. Two papers employed a retinal camera in a rural setting to capture images and send them for offsite evaluation at an urban center [34,36]. These systems identified cataracts in 18–21% of patients. One author proposed a visual function questionnaire to assess cataracts in the context of their impact on patients’ activities of daily living (e.g., personal care, mobility and social interaction, and income generation). Three papers evaluated the costs of their devices, reporting cost reductions between 37–94% compared to commercial direct ophthalmoscopes used in the US, UK and New Zealand [15,30,31].

Seven out of twelve original studies tested new cataract screening tools with non-ophthalmologist personnel, including nurses, primary care providers, community health workers, optometrists, pediatricians, students or even community healers [15,30,31,34,36–39]. For example, John et al. and Katibeh et al..employed non-ophthalmologist eye care workers to capture images which were then shared with offsite ophthalmologists to provide eye care in rural areas [40].

Eight out of twelve original papers also developed computerized processes and tele-health solutions for cataract evaluation in LMICs [41]. Again, John et al. and Katibeh et al. proposed a real-time fundus image sharing system between a rural screening site and offsite providers in urban hospitals for live evaluation by an ophthalmologist [36,40]. Other authors created algorithms to automatically detect and grade cataracts using slit-lamp, retinal, or smartphone camera images, including another study using photos taken at an eye camp [32–35,37,41]. Using their AI platform, Wu et al. estimated a projected 10x increase in the number of cataracts able to be evaluated [42].

Only five of twelve original studies we identified proposed a new cataract evaluation method that was actually developed in a LMIC: the 1999 study in Malawi developing a visual function questionnaire [43] and the four studies in India and Iran that tested their image capture and 2referral/analysis systems in rural screening programs such as eye camps [33,36,37,40]. The remainder of the studies, while not originating from LMICs, developed their tools with the intention of usage in LMICs or rural areas of their country (i.e., China).

## Discussion

Based upon our literature review and analysis, there have been several successful interventions in cataract screening in LMICs. The most common involve a combination of 1) affordable, portable screening devices, 2) screening by local non-ophthalmologist personnel, and 3) tele-ophthalmology approaches that allow for automated data analysis or offsite evaluation of images by ophthalmologists.

The high costs of standard diagnostic tools for cataracts in high-income countries pose a major challenge in resource-limited countries as direct ophthalmoscopes can cost hundreds in USD, and slit lamp biomicroscopes cost in the thousands of USD [15,30,31]. Several authors attempted to work around this barrier using low-cost, portable devices. Notably, the usage of mobile cameras that may be available in patients’ own households could allow for home monitoring of cataract progression [32,33,35]. Studies using smartphone cameras to capture fundus images and evaluate posterior pole pathologies such as diabetic retinopathy, age-related macular degeneration, and glaucoma have already shown a high degree of concordance with traditional methods [44]. Imaging modalities such as retinal cameras can also be extrapolated from other ophthalmic subspecialties to diagnose cataracts [34]. Visual function questionnaires can qualitatively assess cataracts in the context of their impact on patients’ activities of daily living. These surveys have been tested and validated across multiple countries with promising applications in LMICs [43,45], but the precise sensitivity and specificity compared with other methods remains ill-defined. Especially in settings where health literacy may be lower, conducting a visual assessment in language that is meaningful to patients is critical, as personal capabilities and subjective disability may be more important to patients than absolute visual acuity. In addition to being a tool that patients can understand and relate to, surveys also have the added benefit of increasing awareness of visual problems and their treatability.

Cataract screening in settings with little to no access to tertiary or even secondary eye care services may also benefit from initial screening with local primary care providers or mid-level workers such as optometrists, nurses, and technicians [15,30,31,34,36–39]. Broadening the personnel that can serve as a first point of contact for patients in the cataract referral pathway would likely increase the rate at which patients in need of surgical intervention could receive it. Further, Pon et al. reported that 85% of pediatric congenital cataracts are already identified by a primary care provider, so empowering this group of providers through education and accessible devices may also be cost-effective in increasing screening rates and diagnostic accuracy [31].

Aside from the traditional clinical environment, screening may also involve outside initiatives such as the eye camps commonly seen in South and Southeast Asia which provide mass screenings to hundreds or even thousands of patients. These camps, which are coordinated in conjunction with the local government, then offer a referral or second opinion from an ophthalmologist that defines the final diagnosis and interventions necessary. Resources such as imaging systems may be donated by ophthalmic technology companies, as in the case of the Khmer Sight Foundation, a charity aiming to deliver sustainable eye care in Cambodia through educational and physical infrastructure supported by international aid [46]. However, the success and design of such initiatives depends on the context, such as whether they are better implemented as a mobile eye unit to reach multiple locations or a static site with more reliable resources [46]. Additionally, while eye camps have been highly successful in Asia, they rely on being well-publicized, located in densely populated areas, and cultural familiarity or acceptance [39,46]. Meanwhile, a 1994 study cites a total of less than 500 ophthalmologists in Africa, 150 of which are located in South Africa. This implies a ratio of 1 ophthalmologist:1.3 million people, with many countries without a single ophthalmologist [39]. In this case, eye camps would likely be less successful due to the sparse spread of patients compared to ophthalmic providers. In general, several studies have attributed the success of eye care systems in LMICs, including from a cost perspective, to local mid-level workers [4,17,39]. One 2012 study even postulated that reductions in low vision in Timor-Leste over a 5-year period was partially attributed to initiatives aiming to increase the number of mid-level eye care workers, in addition to rural outreach [47]. Such rapid-assessment approaches executed by locally available personnel have been implemented in multiple subspecialties of ophthalmology, dating back to as early as 1990 [48]. However, studies in LMICs have shown limited ability to correctly recognize cataracts in non-ophthalmology primary care workers, necessitating appropriate training and education in order to leverage this group [19].

Tele-health and automated cataract evaluation algorithms including artificial intelligence (AI) can also supplement efforts to increase access to and volume of cataract screening in LMICs where secondary and tertiary eye care services are not readily accessible [41,44]. These approaches bypass the need for interpretation by mid-level workers entirely, solely relying on a technician to capture the initial image, which can then be automatically assessed and flagged for referral to an ophthalmologist [36]. Additionally, one randomized controlled trial deploying a community telehealth screening program in Iran demonstrated a statistically significant increase in eye care utilization after screening, suggesting that telehealth can serve as an effective referral pipeline to engage more patients in the healthcare system [36]. The sheer volume of data utilized by these methods could also be used to establish new cataract grading systems based on global standards or refine existing ones [49] to best serve a broader population. Many successful advances in AI have been developed for analysis of posterior segment pathologies. However, relatively fewer exist for cataract, refractive, and corneal applications, likely because of more limited availability of anterior segment image databases most of which originate from the United States, Europe, or China [44,50]. The application of algorithms based on patient data from high-income countries into low-resource settings remains a major ethical challenge. The AI algorithms we identified were developed using data from Chinese hospitals [32,35,51], with the exception being Devaraj et al. in India [37], suggesting that the initial development of these algorithms may require large datasets that are easier to acquire in densely populated urban settings. The eventual acquisition and integration of broader global data would only further improve the accuracy, generalizability, and validation of these methods, which were some of the major challenges of the earlier AI-based models [50,52]. Given the limited infrastructure for health data in low-resource settings, the aforementioned low-cost and/or portable screening devices such as readily accessible smartphones that can generate images for AI or telehealth analysis only become more critical in making technology more equitable worldwide.

Several other barriers hinder the implementation of AI in clinical practice, including data privacy and ethical concerns, legal and proprietary constraints, and variability in image quality and formatting [50]. Most current AI models are trained to detect specific pathologies – such as diabetic retinopathy or early glaucomatous changes – and may overlook conditions for which they were neither trained nor intended to detect [53]. While efforts are underway to develop generalizable disease detection models, these have yet to achieve widespread clinical integration [54]. In addition, sustainability and climate change present further ethical considerations. AI systems require substantial computational power, which contributes to increased energy consumption and carbon emissions [55]. These concerns are especially pertinent in low-resource settings, where infrastructure may be limited and consistent access to electricity cannot be guaranteed.

Regarding the potential economic impacts of AI and telehealth, Wu et al. advocate for a hierarchical screening model in both urban and rural China. This system is comprised of an initial AI-based home-based screening of images captured at home, followed by referral of patients with suspected pathology to a community facility to receive AI-assisted diagnosis, with final referral of patients needing surgical care to tertiary hospitals. They demonstrated that this screening method was more cost-effective than telehealth screening or AI screening alone, with estimated lifetime costs of up to $2,737 and up to 14.3 quality-adjusted life years gained per person cost [56]. However, more detailed investigation of expenses related to platform development and/or management, Internet access, any screening tools involved, and staff training would provide valuable insight into how these technologies can be responsibly integrated into resource-limited settings.

While this paper focuses on identifying patients in need of cataract surgery, expanding cataract surgical rates (CSR) in low-resource settings requires an understanding of their ability to expand surgical capacity and ensure good surgical outcomes. Increasing CSR relies on sufficient and affordable staffing, infrastructure, equipment, and ability to follow-up [28]. It has been shown that CSR in a country generally correlates with, but is not absolutely predicted by, the gross domestic product per capita, likely due to inequitable distribution of health services between rural and urban areas within a country [5]. Most of the costs related to cataract surgery are related to personnel and facilities, with the remaining 20% representing consumable supplies such as eye drops and intraocular lenses. Improving the distribution of health facilities between rural and urban areas as well as creating specialist outreach or referral programs is critical in sustainably improving care for underserved populations. Drawing on previous efforts to improve access to surgical care in general, lack of skilled health personnel in low-resource settings could be addressed using financial incentives or by creating training programs in LMICs or rural areas. Similarly to our findings, existing community health workers can also be recruited and trained as surgical staff, including task shifting in which non-ophthalmologist personnel (i.e., nurses) are trained to perform cataract surgery. However, the use of nonphysicians to perform cataract surgery is controversial and would likely only be useful in areas with extremely low densities of ophthalmologists, as in many African countries, and comparisons of surgical outcomes would be critical [57]. The usage of surgical techniques that do not require expensive phacoemulsification equipment (such as manual small-incision cataract surgery and extracapsular cataract extraction) and low-cost, locally manufactured lenses can improve the cost-effectiveness of cataract surgery to as low as $15.68 per operation, including fixed costs [57]. Lastly, costs of surgery to patients can be reduced (i.e., through “tiered pricing” based on the paying capacity of different patient populations), waived (i.e., through conditional cash transfers specifically for health services), or integrated into general health plans. These measures have been shown to increase usage of surgical health services, but their efficacy specifically in the context of cataract surgery remains unstudied [57,58].

Lack of data remains the main barrier to expansion of cataract services in low-resource settings, and the existing research is also insufficient to influence policy. In our study, sparse and/or varied reports of the cost and efficacy of various interventions prevented us from providing robust comparisons between methods for cataract diagnosis. The consensus is that there still remains a significant need for strengthened health information systems to monitor eye care services and surgical outcomes, capacity to initiate and carry out research, and pipelines to translate research to policy and practice [59]. The United Nations General Assembly’s Vision For Everyone resolution projects that these goals be addressed in order to achieve their Sustainable Development Goals by 2030 [60]. Such research should focus on the ability of health care systems to adopt the aforementioned interventions, their financial burden, and their impact on surgical access rates and outcomes [58,59]. Eye health research in low-resource settings is also likely to remain under-resourced, necessitating collaboration with other countries for funding, development of health technologies and systems, and research [59].

Despite all these advances, increasing cataract surgical output through affordable technologies and improved health systems will not succeed without tandem efforts to improve education surrounding cataracts & vision loss. Many studies suggest that significant barriers to surgery include lack of knowledge of the treatability of cataracts, lack of trust in surgical interventions and/or a preference for natural remedies, lack of accessible, affordable, reliable transportation, or beliefs that vision loss is inevitable [5,15,16,20,21,46,61,62]. It should also be noted that only a small number of original studies proposing methods for cataract screening in low-resource settings were actually developed in LMICs [15,40,43]. Thus, future research would benefit from collaborative efforts with researchers and providers in those target countries to best align new interventions with the specific needs, values, and healthcare systems in those populations.

## Conclusions

In summary, improving cataract diagnosis in low- and middle-income countries may rely on a multifaceted approach involving low-cost tools, including those from other ophthalmic subspecialties, utilized by local personnel and augmented by automated analysis methods and/or tele-ophthalmology. Such interventions may result in increased screening rates and earlier identification of and intervention on operable cataracts, at a reduced cost compared to having visiting ophthalmologists. Along with limited screening, limited surgical capacity in such regions remains a major setback to decreasing blindness caused by cataracts. In parallel to system-level changes, improving patients’ knowledge of the treatability of cataract and addressing cultural beliefs surrounding vision loss and eye care are critical to the holistic care of patients in low-resource settings. Finally, innovations to reduce disparities in cataract diagnosis and surgery must be supported by efforts to improve health systems and increase their capacity for data collection and disease monitoring, research, and practical implementation of new initiatives.
